# Supplementary material for: Thrombopoietin mutation in congenital amegakaryocytic thrombocytopenia treatable with romiplostim
Source: EMBO Mol Med. 2017 Nov 30;10(1):63–75. doi: 10.15252/emmm.201708168 (PMC5760853; doi:10.15252/emmm.201708168)
Supplement: Supplementary file 1 — Appendix [file EMMM-10-63-s001.pdf]

**Manuscript "Thrombopoietin mutation in congenital amegakaryocytic thrombocytopenia treatable with romiplostim"** by Pecci et al.

**APPENDIX**

**TABLE OF CONTENTS**


**Appendix Table S1.** Exact *P*-values for data reported in Figure 2

**Appendix Table S2.** Exact *P*-values for data reported in Figure 3

**Appendix Table S3.** Exact *P*-values for data reported in Figure 4

**Appendix Table S4.** Exact *P*-values for data reported in Figure 5

**Appendix Table S5.** Exact *P*-values for data reported in Figure 6

**Appendix Table S1. Exact *P*-values for data reported in Figure 2**

| **Data reported in Panel B** | | |
|---|---|---|
| | | ***P*-VALUE** |
| **FLAG/Actin ratio (a.u.)** | p.R119C compared to WT | 0.0014 |
| | p.R38C compared to WT | 0.0195 |
| **THPO/Actin ratio (a.u.)** | p.R119C compared to WT | 0.0482 |
| | p.R38C compared to WT | 0.0136 |
| **Data reported in Panel D** | | |
| | | ***P*-VALUE** |
| **FLAG/Actin ratio (a.u.)** | p.R119C compared to WT 24h | 0.0016 |
| | p.R119C compared to WT 48h | 0.0693 |
| | p.R38C compared to WT 24h | 0.0033 |
| | p.R38C compared to WT 48h | 0.0082 |
| **THPO/Actin ratio (a.u.)** | p.R119C compared to WT 24h | 0.0206 |
| | p.R119C compared to WT 48h | 0.0431 |
| | p.R38C compared to WT 24h | 0.0152 |
| | p.R38C compared to WT 48h | 0.0153 |

**Appendix Table S2. Exact *P*-values for data reported in Figure 3**

| | | ***P*-VALUE** |
|---|---|---|
| **Cell proliferation with THPO-conditioned media 0.5 µl** | p.R119C compared to WT | 0.00019 |
| | p.R38C compared to WT | 0.0014 |
| **Cell proliferation with THPO-conditioned media 1.0 µl** | p.R119C compared to WT | 0.0005 |
| | p.R38C compared to WT | 0.0005 |

**Appendix Table S3. Exact *P*-values for data reported in Figure 4**

| Data reported in Panel B | | | |
|---|---|---|---|
| | | | ***P*-VALUE** |
| **Relative protein THPO/GAPDH (%)** | WT CHX compared to WT DMSO | 8h | 8.800E-05 |
| | | 24h | 0.012 |
| | | 48h | 0.0006 |

**Appendix Table S4. Exact *P*-values for data reported in Figure 5**

| | | ***P*-VALUE** |
|---|---|---|
| **Cell proliferation with THPO 60 pg/mL** | p.R119C compared to WT | 0.0175 |
| | p.R38C compared to WT | 0.0146 |
| **Cell proliferation with 120 pg/mL of THPO** | p.R119C compared to WT | 0.0162 |
| | p.R38C compared to WT | 0.0218 |
| **Cell proliferation with 240 pg/mL of THPO** | p.R119C compared to WT | 0.0043 |
| | p.R38C compared to WT | 0.0012 |

**Appendix Table S5. Exact *P*-values for data reported in Figure 6**

| **Data reported in Panel B** | | | |
|---|---|---|---|
| | | | ***P*-VALUE** |
| **pSTAT5/STAT5** | **THPO-conditioned supernatant 0.5 µl** | p.R119C compared to WT | 0.0001 |
| | | p.R38C compared to WT | 0.0001 |
| | **THPO 240 pg/mL** | p.R119C compared to WT | 0.0002 |
| | | p.R38C compared to WT | 0.014 |
| **pAKT/AKT** | **THPO-conditioned supernatant 0.5 µl** | p.R119C compared to WT | 0.009 |
| | | p.R38C compared to WT | 0.005 |
| | **THPO 240 pg/mL** | p.R119C compared to WT | 0.040 |
| | | p.R38C compared to WT | 0.011 |
| **pERK/ERK** | **THPO-conditioned supernatant 0.5 µl** | p.R119C compared to WT | 0.007 |
| | | p.R38C compared to WT | 0.009 |
| | **THPO 240 pg/mL** | p.R119C compared to WT | 0.024 |
| | | p.R38C compared to WT | 0.030 |
