# Supplementary material for: Thrombopoietin mutation in congenital amegakaryocytic thrombocytopenia treatable with romiplostim
Source: EMBO Mol Med. 2017 Nov 30;10(1):63–75. doi: 10.15252/emmm.201708168 (PMC5760853; doi:10.15252/emmm.201708168)
Supplement: Supplementary file 2 — Expanded View Figures PDF [file EMMM-10-63-s002.pdf]

Expanded View Figures

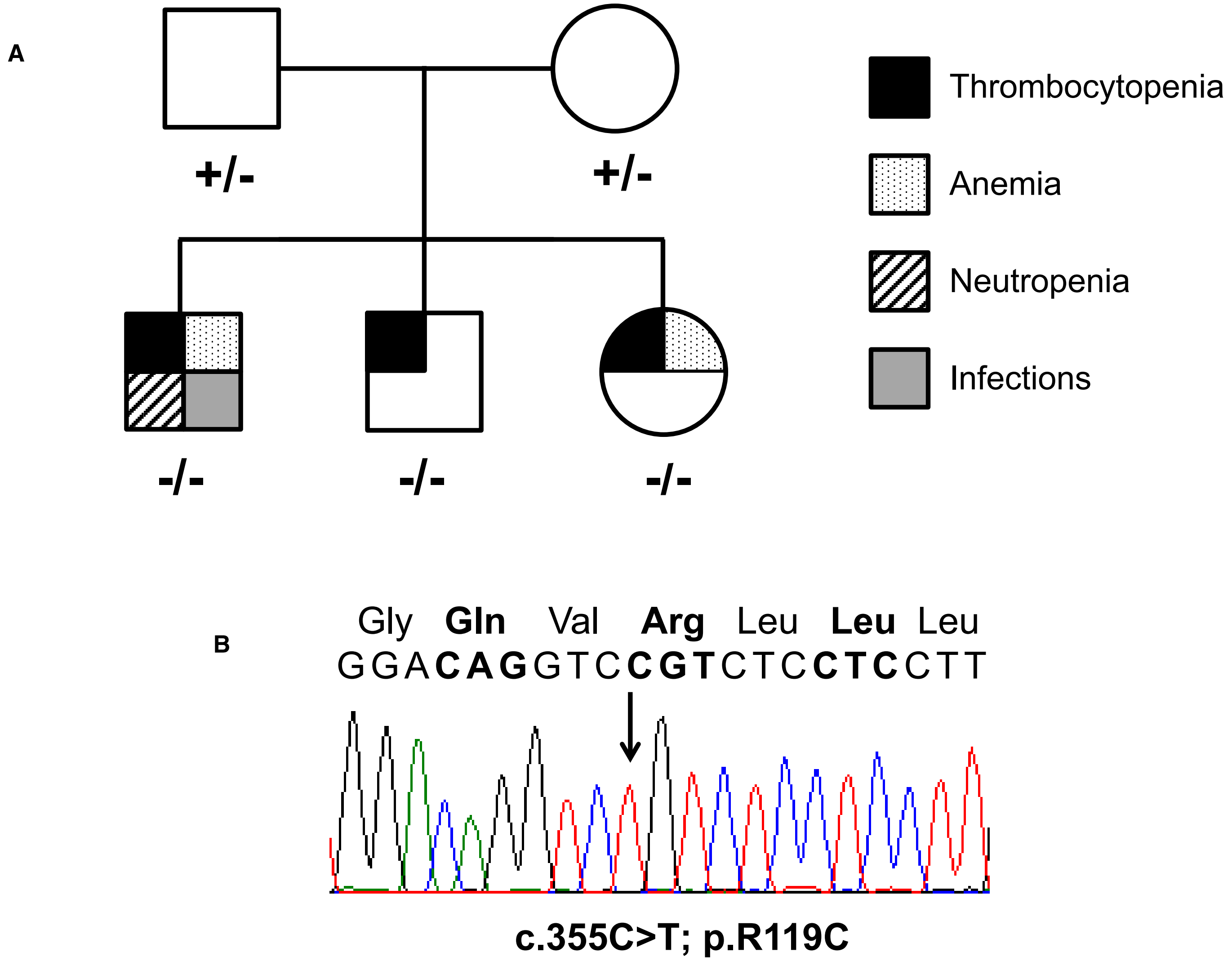

Figure EV1. Identification of the p.R119C mutation of THPO.

A Family pedigree indicating the genotype and phenotype of the family members. "+" and "–" indicate the wild-type and the mutant (c.355C>T; p.R119C) allele, respectively. Different symbols indicate the presence of severe thrombocytopenia, anemia, neutropenia, or infections, as shown in the figure.

B Electropherogram showing the homozygous c.355C>T mutation in the proband.

© 2017 The Authors

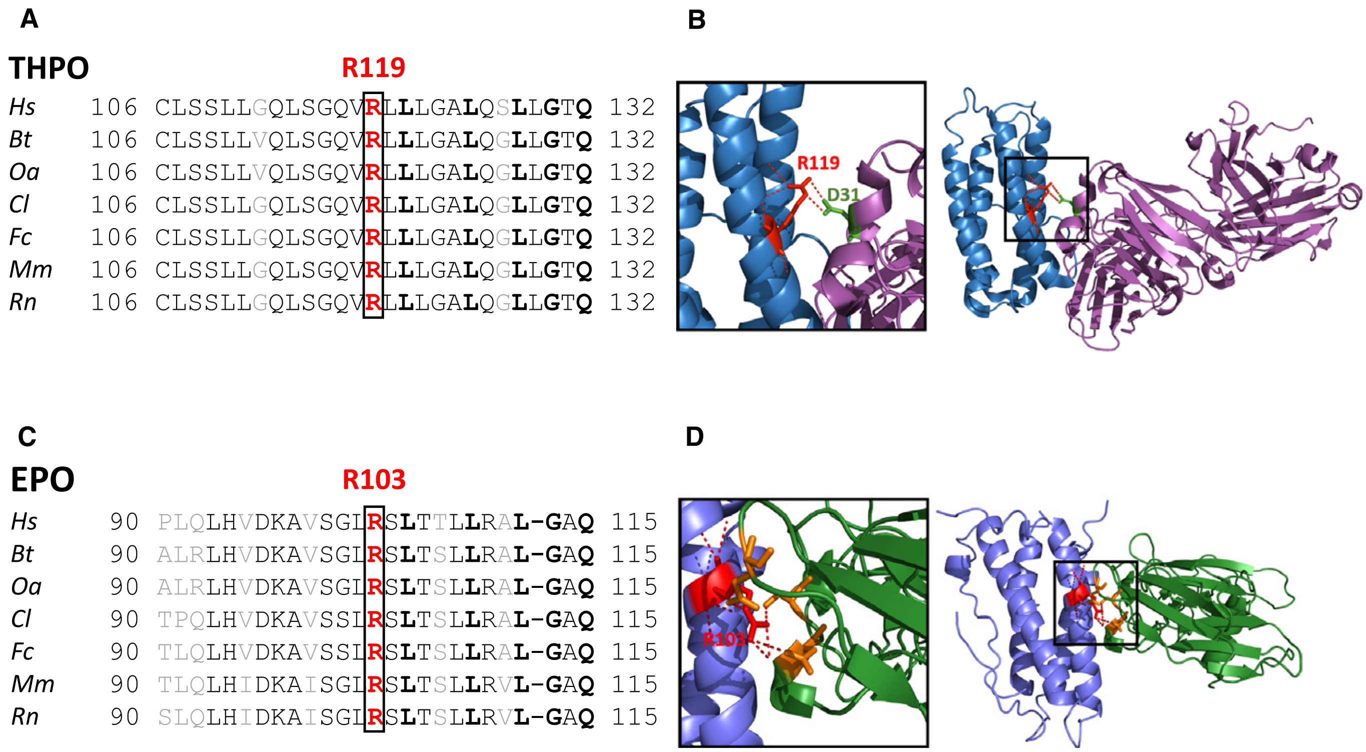

Figure EV2. R119 is highly conserved, and the p.R119C is expected to affect THPO binding to its receptor.

A Multiple-sequence alignment analysis of THPO orthologs from different species. *Hs*, *Homo sapiens* (NP_000451.1); *Bt*, *Bos taurus* (NP_001159512.1); *Oa*, *Ovis aries* (XP_011984347.1); *Cl*, *Canis lupus familiaris* (XP_005639837.1); *Fc*, *Felis catus* (NP_001157128.1); *Mm*, *Mus musculus* (NP_033405.1); *Rn*, *Rattus norvegicus* (NP_112395.1). Residues in bold are conserved among both THPO and EPO orthologs. Residues in gray are not conserved within THPO or EPO.

B Cartoon representation of the structure of the receptor binding domain of THPO (in sky blue) in complex with a neutralizing antibody fragment (in magenta) (PDB 1V7M) (Feese *et al*, 2004). The inset shows a zoom of the interaction involving R119 (in red) with the D31 of the antibody fragment (in green).

C Multiple-sequence alignment analysis of EPO orthologs from different species. *Hs*, *Homo sapiens* (NP_000790.2); *Bt*, *Bos taurus* (NP_776334.1); *Oa*, *Ovis aries* (NP_001019908.1); *Cl*, *Canis lupus familiaris* (NP_001006647.1); *Fc*, *Felis catus* (NP_001009269.1); *Mm*, *Mus musculus* (NP_031968.1); *Rn*, *Rattus norvegicus* (NP_058697.1). Residues in bold are conserved among both THPO and EPO orthologs. Residues in gray are not conserved within THPO or EPO.

D Cartoon representation of the structure of the receptor binding domain of EPO (in violet) in complex with its receptor (in green) (PDB 1EER) (Syed *et al*, 1998). The inset shows a zoom of the cluster salt bridge interactions involving R103 (in red), the analogous of R119 in THPO.
